# COVID-19 and chronic kidney disease: a bibliometric analysis

**DOI:** 10.1097/MS9.0000000000001640

**Published:** 2023-12-19

**Authors:** Wenze Jiang, Yuting Chen, Yuxin Zhao, Yang Gao, Tianyang Cheng, Enhui Qian, Yating Hou, Keda Lu

**Affiliations:** a First Clinical Medical College; b Third Clinical Medical College, Zhejiang Chinese Medical University; c Third Affiliated Hospital of Zhejiang Chinese Medical University, Hangzhou, China

**Keywords:** bibliometric analysis, CiteSpace, COVID-19, chronic kidney disease

## Abstract

**Background:**

The COVID-19 pandemic has caused over 656 million confirmed cases and over 6.6 million deaths worldwide. Chronic kidney disease (CKD) is considered a high-risk factor for COVID-19; therefore, considerable research has been conducted in this field. Therefore, this study aims to conduct a bibliometric analysis of publications related to COVID-19 and CKD.

**Methods:**

Publications were retrieved from the Web of Science Core Collection database on 16 January 2023 and screened based on inclusion criteria. Then the authors used Microsoft Excel and CiteSpace to analyze the included publications from the following seven aspects: countries/regions, institutions, journals, authors, cited references, and keywords.

**Results:**

In total, 622 publications were included in the study. The USA has the most publications in this field, followed by China. The Icahn School of Medicine at Mount Sinai and Harvard Medical School had the highest number of publications in the field. Journal of Clinical Medicine had the largest number of publications, and Lancet was the most cited journal. Alberto Ortiz was the author with the largest number of publications, but there were no influential authors in this field. The highly cited references are mainly clinical studies on COVID-19. Research hotspots in this field include end-stage recent disease, cardiovascular disease, kidney metastasis, diabetes Mellitus, acute kidney injury, meta-analysis, and consistent plasma.

**Conclusions:**

The USA, China, and some European countries and their institutions are major contributors to these publications. End-stage renal disease, acute kidney injury, kidney transplantation and convalescent plasma are current hot topics in the field.

## Introduction

Highlights

- This study is to use bibliometrics to summarize the research on COVID-19 and chronic kidney disease.
- The United States has contributed the most to research related to COVID-19 and chronic kidney disease.
- COVID-19 has had a profound impact on renal replacement therapy, especially renal transplantation, and deserves further in-depth investigation.
- The research frontier in this field of COVID-19 and chronic kidney disease may focus on solving clinical problems.

The SARS-CoV-2 virus, which causes COVID-19, has significantly influenced human health on a global scale. On 11 March 2020, COVID-19 was declared a pandemic by the WHO^1^. According to WHO, over 656 million confirmed cases of COVID-19 and over 6.6 million deaths have been reported globally as of 1 January 2023^2^.

Although COVID-19 primarily damages the lungs, it can also affect the kidneys through a variety of mechanisms^3^. SARS-CoV-2 affects the kidney through the high expression of angiotensin-converting enzyme 2, transmembrane serine protease 2, and tissue proteinase L in the kidney^4^. Studies have shown that patients with chronic kidney disease (CKD) and COVID-19 have an increased risk of death and hospitalization^5^.

Bibliometric analysis is a method of objective and mathematical analysis of scientific publications related to a specific matter^6^. Bibliometrics differs from traditional literature reviews in that it focuses on the institution, authorship, and social structure of the literature^7^. This unique approach saves researchers significant time and effort by extracting reliable information from large quantities of literature. Since the pandemic, studies on COVID-19 have been conducted extensively in the area of CKD. However, there is a lack of bibliometric analysis to evaluate the associated literature comprehensively, summarize the latest trend, and predict research hotspots. This study aimed to perform an in-depth bibliometric analysis on this subject in order to provide policymakers, clinicians, researchers, and the general public an invaluable reference.

## Methods

For this bibliometric analysis, the literature data were obtained from the Web of Science Core Collection (WoSCC) database. Deviations caused by daily database renewal were prevented by retrieving and downloading all documents published from the WoSCC database on 16 January 2023. The search terms were: TOPICS = (COVID-19 OR Coronavirus-19 OR SARS-CoV-2 OR novel coronavirus OR 2019-nCoV) AND (CKD OR chronic kidney disease OR chronic renal disease OR chronic renal insufficiency OR chronic kidney insufficiency). Documents defined as original articles and review articles were included, while other document types, such as letters, guidelines, and meeting abstracts, were excluded from this analysis. The language was limited to English. The contents of the literature were examined one by one. Literature not related to COVID-19 and CKD was excluded from the study. The data were downloaded using the “export” function, and the “record content” was set to “full record and cited references”. The data was saved in plain text and named in download_.txt format.

Then, we imported the data to Microsoft Excel 2016 and CiteSpace(6.1.6) for further analysis. Microsoft Excel 2016 was used to create tables showing the type and number of publications, the top 10 countries/regions, institutions, journals, and authors regarding the number of publications, and the top 20 publications ranked by the citations. CiteSpace is a popular bibliometric visualization tool used for data processing and visualization^8^. In this study, we used CiteSpace to construct a visual knowledge graph of countries/regions, institutions, journals, authors, cited references, and keywords.

## Results

### Included publications

**Figure 1:**
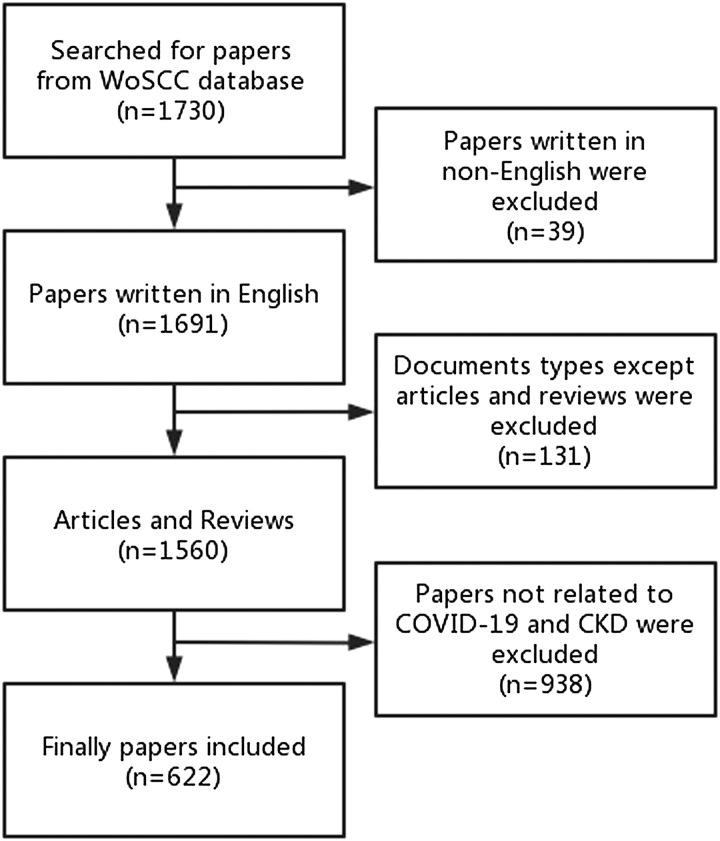
Publication screening flow chart. CKD, chronic kidney disease; WOSCC, Web of Science Core Collection.

Initially, 1730 publications were obtained from the WoSCC database. According to the inclusion and exclusion criteria, a total of 622 publications, including 499 articles and 123 reviews, were eventually included. See Figure 1 for the search process.

### Analysis of countries/regions

To comprehend which countries/regions made significant contributions to the research of COVID-19 and CKD, the study analyzed the number of publications from 85 countries/regions. The greatest number of publications came from USA (151, 24.28%), followed by China (74, 11.90%), Italy (73, 11.74%), and Spain (56, 9.00%).

In CiteSpace, betweenness centrality is used to find and measure the importance of documents, and the nodes with betweenness centrality greater than 0.1 are the key points^8^. An analysis of the country distribution of these publications shows that there are four countries/regions whose betweenness centrality greater than 0.1, and they are USA (0.37), England (0.25), Canada (0.17), and Spain (0.12).

**Figure 2:**
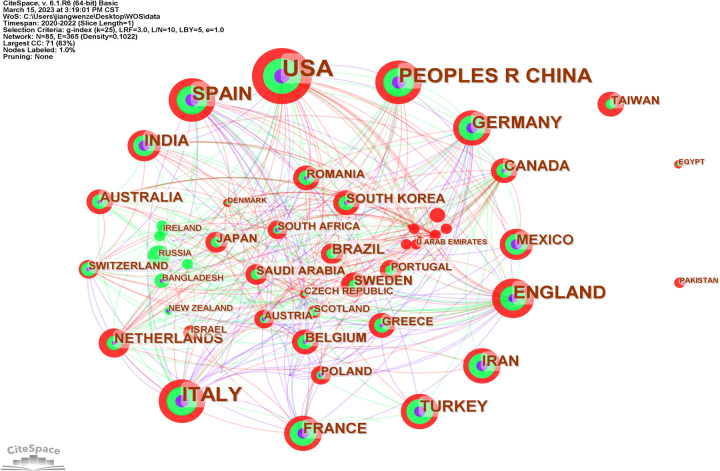
The country co-authorship network of COVID-9 and chronic kidney disease.

As illustrated in Figure 2, each circle represents a country/region, and the size of the circle indicates the number of publications output by the country/region. The lines between the circles denote cooperation between countries/regions, with wider lines, and closer cooperation. Figure 2 shows active cooperation between many countries, such as USA, China, Spain, Italy, and England. However, Taiwan, Egypt, and Pakistan did not cooperate with other countries/regions.

### Analysis of institutions

A total of 622 publications in the field were distributed among 3579 institutions, and the strength of the partnership varies between countries and between institutions. The top 10 institutions with the highest number of publications related to COVID-19 and CKD research are listed in Table 1. Icahn School of Medicine at Mount Sinai and Harvard Medical School were the institutions with the highest number of publications in the field, and University of Milan was the institution with the highest betweenness centrality. Among the top 10 institutions, 5 institutions were from USA and the other five were from Italy, China, Turkey, the Netherlands, and England.

**Table 1:** The top 10 institutions in terms of the number of publications in COVID-19 and CKD.

Rank	Institution	Publication	Country	BC
1	Icahn School of Medicine at Mount Sinai	15	USA	0.20
2	Harvard Medical School	15	USA	0.16
3	University of Milan	13	Italy	0.22
4	Stanford University	13	USA	0.12
5	Huazhong University of Science and Technology	12	China	0.00
6	University of Health Sciences	10	Turkey	0.08
7	Brigham and Women’s Hospital	10	USA	0.03
8	University College London	9	England	0.14
9	University of Groningen	9	Netherlands	0.06
10	University of Washington	9	USA	0.02

BC, betweenness centrality; CKD, chronic kidney disease.

**Figure 3:**
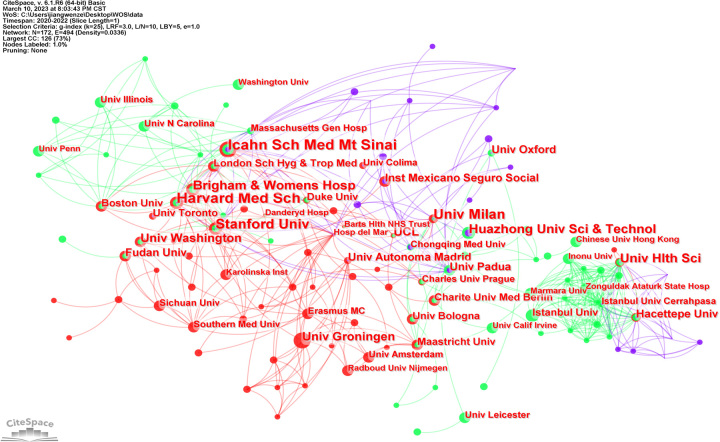
The institution co-authorship network of COVID-9 and chronic kidney disease.

As seen in Figure 3, the network map of institutional co-authorship was created. We found close collaboration between different institutions, and institutions from the same country collaborated more closely with each other due to their geographical advantage. The research institutions from USA were key nodes in the collaborative network, such as Harvard Medical School, Stanford University, and the Icahn School of Medicine at Mount Sinai.

### Analysis of journals and cited journals

From 2020 to 2022, 622 papers on COVID-19 and CKD research were published in 285 journals. From Table 2, we can see that the journal with the highest number of publications was Journal of Clinical Medicine, followed by PLoS One. The impact factor is an internationally accepted journal evaluation index. In general, the higher the impact factor, the greater the impact of the journal. Clinical Infectious Diseases had the highest impact factor among the top 10 journals.

**Table 2:** The top 10 journals in terms of the number of publications relating to COVID-19 and CKD.

Rank	Journal	Publication	Country	IF (2021)	Citations	JCR (2022)
1	Journal of Clinical Medicine	25	USA	4.964	116	Q1
2	PLoS One	20	USA	3.752	236	Q2
3	Clinical Kidney Journal	18	England	5.86	108	Q1
4	Frontiers in Medicine	17	Switzerland	5.058	61	Q1
5	Vaccines	16	Switzerland	4.961	31	Q2
6	Journal of Nephrology	11	Italy	4.393	96	Q2
7	Nephrology Dialysis Transplantation	10	England	7.186	219	Q1
8	Blood Purification	9	Switzerland	3.348	49	Q3
9	Scientific Reports	8	England	4.996	111	Q1
10	Clinical Infectious Diseases	8	USA	20.999	173	Q1

CKD, chronic kidney disease; IF, impact factor; JCR, Journal Citation Reports.

Table 3 shows the top 10 cited journals in terms of the number of publications relating to COVID-19 and CKD. All the top 10 journals are from USA or England, and all journals except PLoS One belong to the Q1 region. Three journals in Table 3 have an impact factor of over 100, with Lancet having the highest IF, followed by New England Journal of Medicine and JAMA-Journal of the American Medical Association.

**Table 3:** The top 10 cited journals in terms of the number of citations relating to COVID-19 and CKD.

Rank	Journal	Citations	Country	IF (2021)	JCR (2022)
1	Lancet	409	England	202.731	Q1
2	New England Journal of Medicine	399	USA	176.079	Q1
3	Kidney International	344	USA	18.998	Q1
4	JAMA-Journal of the American Medical Association	340	USA	157.335	Q1
5	Journal of the American Society of Nephrology	261	USA	14.978	Q1
6	PLoS One	236	USA	3.752	Q2
7	Nature	222	England	69.503	Q1
8	Nephrology Dialysis Transplantation	219	England	7.186	Q1
9	American Journal of Kidney Diseases	209	USA	11.072	Q1
10	Bmj-British Medical Journal	200	England	93.333	Q1

CKD, chronic kidney disease; IF, impact factor; JCR, Journal Citation Reports.

### Analysis of authors

The included 622 publications were produced by 5826 authors, and the top 10 authors with the highest number of publications in COVID-19 and CKD research are shown in Table 4. Alberto Ortiz, from Spain, is the most published author in this field, followed by Ron T Gansevoort, from Netherlands. The top 10 authors all have an H-index greater than 10, with Kamyar Kalantar-Zadeh having the highest H-index (115). From Table 4, we can see that there is no author with betweenness centrality greater than 0.1, which suggests that no influential authors have emerged in COVID-19 and CKD research.

**Table 4:** The top 10 authors with the highest number of publications in COVID-19 and CKD research.

Rank	Author	Frequency	BC	H-index	Country
1	Ortiz, Alberto	12	0.04	25	Spain
2	Gansevoort, Ron T	6	0.04	86	Netherlands
3	D’marco, Luis	5	0.03	14	Spain
4	Dolff, Sebastian	5	0	22	Germany
5	Cozzolino, Mario	5	0	42	Italy
6	Ciceri, Paola	5	0	16	Italy
7	Galassi, Andrea	5	0	18	Italy
8	Crespo, Marta	4	0.03	15	Spain
9	Arici, Mustafa	4	0.01	30	Turkey
10	Kalantar-zadeh, Kamyar	4	0	115	USA

BC, betweenness centrality; CKD, chronic kidney disease.

**Figure 4:**
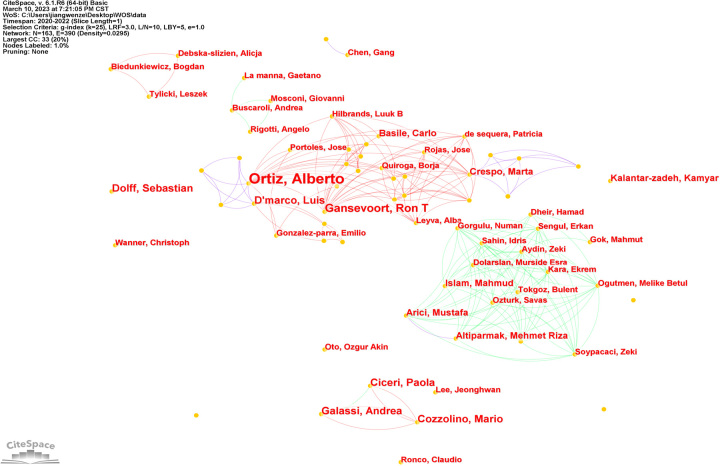
The author co-authorship network of COVID-9 and chronic kidney disease.

Figure 4 shows that there is a cooperative network between different authors. The result shows that there were several groups of authors, and there was an extensive collaboration among authors of the same group. Some authors were key figures in collaborative research in the field, such as Alberto Ortiz, Ron T Gansevoort, and Marta Crespo.

### Analysis of cited references

A total of 248 publications were cited more than five times in the included publications, and the top 10 publications were listed in Table 5. The top 10 publications were all published in 2020, and their betweenness centrality were all less than 0.1. The most cited reference, “Clinical course and risk factors for mortality of adult inpatients with COVID-19 in Wuhan, China: a retrospective cohort study^9^”, was published with 172 citations in Lancet by Zhou F *et al.* ^9^ This paper reported a retrospective cohort study involving 191 laboratory-confirmed COVID-19 patients, which was the largest study on patients diagnosed with COVID-19 at that time.

**Table 5:** The top 10 cited references in terms of the number of citations relating to COVID-19 and CKD.

Rank	Cited references	Citations	BC	Year
1	Clinical course and risk factors for mortality of adult inpatients with COVID-19 in Wuhan, China: a retrospective cohort study	172	0.07	2020
2	Kidney disease is associated with in-hospital death of patients with COVID-19	138	0.05	2020
3	Factors associated with COVID-19-related death using OpenSAFELY	108	0.02	2020
4	Clinical features of patients infected with 2019 novel coronavirus in Wuhan, China	105	0.04	2020
5	Clinical Characteristics of Coronavirus Disease 2019 in China	103	0.02	2020
6	Renal histopathological analysis of 26 postmortem findings of patients with COVID-19 in China	75	0.08	2020
7	Acute kidney injury in patients hospitalized with COVID-19	72	0.06	2020
8	Characteristics of and Important Lessons From the Coronavirus Disease 2019 (COVID-19) Outbreak in China: Summary of a Report of 72 314 Cases From the Chinese Center for Disease Control and Prevention	67	0.00	2020
9	Chronic kidney disease is associated with severe COVID-19 infection	66	0.00	2020
10	Presenting Characteristics, Comorbidities, and Outcomes Among 5700 Patients Hospitalized With COVID-19 in the New York City Area	65	0.03	2020

BC, betweenness centrality; CKD, chronic kidney disease.

**Figure 5:**
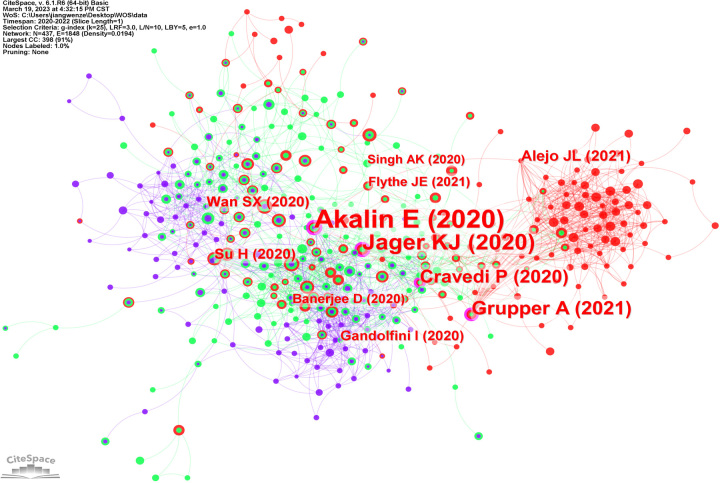
The co-citation network of cited references.

The co-citation network of cited references is presented in Figure 5, the larger nodes represent the more cited times and strong betweenness centrality. In the network, the four largest nodes are easily distinguished by a purple circle. The four publications whose betweenness centrality greater than 0.1 are “Covid-19 and Kidney Transplantation^10^” (0.14), “Results from the ERA-EDTA Registry indicate a high mortality due to COVID-19 in dialysis patients and kidney transplant recipients across Europe^11^” (0.12), “Humoral Response to the Pfizer BNT162b2 Vaccine in Patients Undergoing Maintenance Hemodialysis^12^” (0.1) and “COVID-19 and kidney transplantation: Results from the TANGO International Transplant Consortium^13^” (0.1).

### Analysis of keywords

After merging synonymous keywords, we can get Table 6 lists the top 10 keywords with the highest frequency in COVID-19 and CKD research. Besides chronic kidney disease (166) and COVID-19 (109), other keywords with high frequency in this study include outcome (82), acute kidney injury (81), mortality (75), risk factor (60), risk (54), disease (50), infection (49) and coronavirus (41).

**Table 6:** The top 10 keywords with the highest frequency in COVID-19 and CKD research.

Rank	Keyword	Frequency	Degree	BC
1	Chronic kidney disease	166	14	0.02
2	COVID-19	109	12	0.02
3	Outcome	82	17	0.03
4	Acute kidney injury	81	23	0.05
5	Mortality	75	26	0.08
6	Risk factor	60	10	0.02
7	Risk	54	15	0.02
8	Disease	50	13	0.02
9	Infection	49	14	0.02
10	Coronavirus	41	8	0.01

BC, betweenness centrality; CKD, chronic kidney disease.

**Figure 6:**
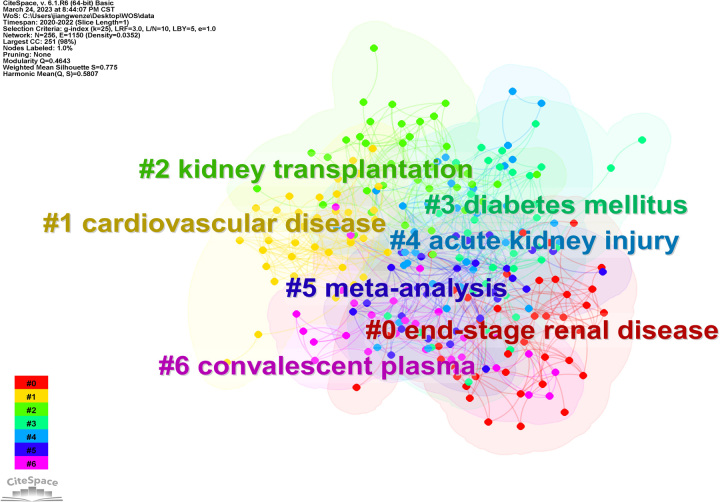
Network of the main keywords’ clusters in the studies of COVID-19 and chronic kidney disease research.

Clusters can be constructed in the network map based on analysis of the co-occurrence of keywords to summarize the research hotspots and derive the fundamental knowledge structure. Figure 6 shows seven clusters of keywords, with each cluster composed of multiple closely related words. These seven clusters are #0 end-stage renal disease, #1 cardiovascular disease, #2 kidney transplantation, #3 diabetes mellitus, #4 acute kidney injury, #5 meta-analysis, and #6 convalescent plasma.

## Discussion

### General information

Over the past 3 years, the COVID-19 epidemic has spread in countries around the world, causing a serious public health disaster. Although countries around the world have invested heavily in tackling COVID-19 and have produced a large number of high-quality studies and publications in a short period, COVID-19 and its relationship with other diseases are still unknown to researchers. Therefore, it is essential to summarize the studies targeting COVID-19 by bibliometric analysis. This can help us to identify landmark papers and high-impact journals, and summarize the research hotspots in the field, indicating future research findings.

The analysis of countries/regions reveals that the USA is the country with the most publications and the strongest influence on COVID-19 and CKD research. This may be because the USA is the country most affected by COVID-19 and has the most advanced medical and scientific capabilities. Although China is second only to the USA in terms of the number of publications, it has a less academic impact. This indicates that China still lacks high-quality research and publications in this field. In the analysis of institutions, the Icahn School of Medicine at Mount Sinai (USA) and Harvard Medical School (USA) were the institutions with the most publications. Five of the top 10 institutions in terms of the number of publications are from the USA, indicating the absolute leadership of the USA in this field. Despite the devastation caused by COVID-19 in Africa, we found that none of the top 10 countries and institutions in this research field are from Africa. This may be related to the difficulties of researchers in Africa in accessing information, research, and publication.

Journal of Clinical Medicine is the most published journal in this research area, followed by PLoS One and Clinical Kidney Journal. Researchers in this field have focused their research on clinical issues arising since the COVID-19 pandemic. Most of the cited journals were high-impact journals, and the journal with the highest number of citations was Lancet, followed by New England Journal of Medicine, indicating that research on COVID-19 and CKD is gaining significant attention worldwide.

Alberto Ortiz from Spain is the author of most publications and has played a major role in the field of COVID-19 and CKD research. “Chronic kidney disease is a key risk factor for severe COVID-19: a call to action by the ERA-EDTA^14^” is the most influential publication of Alberto Ortiz. This article defines CKD as the most prevalent risk factor for severe COVID-19 and advocates the inclusion of patients with CKD in clinical trials to test the efficacy of drugs and vaccines to prevent severe COVID-19^14^.

Kamyar Kalantar-Zadeh is the author with the highest H-index among the top 10 authors in terms of the number of publications. Kalantar-Zadeh’s specific studies include the potential role of lipid mediators in COVID-19 leading to renal injury and fibrosis^15^; developing a quick nutrition guide for COVID-19 patients with CKD^16^; effects of hormonal changes on sarcopenia in COVID-19 patients with CKD^17^ and novel therapeutic approaches for COVID-19 in chronic kidney disease and transplant^18^.

Of the top 10 cited references in terms of the number of citations, nine were clinical studies on COVID-19, indicating that researchers are increasingly focusing on the realities of patients, which is in line with the specific situation we are currently facing. In addition, we found four articles^10–13^ with betweenness centrality greater than 0.1 all related to renal replacement therapy. COVID-19 has had a profound impact on renal replacement therapy, especially renal transplantation, and deserves further in-depth investigation.

### The hotspots and frontiers

Based on keywords co-occurrence analysis, the current research hotspots in COVID-19 and CKD are indicated by these seven clusters. We divided the seven clusters into three categories, #0 end-stage renal disease, #1 cardiovascular disease, #3 diabetes mellitus, and #4 acute kidney injury are diseases of interest in the field, #2 kidney transplantation and #6 convalescent plasma are treatments, and #5 meta-analysis is the primary method of study.

The kidney is an important target of SARS-CoV-2^19^. On the one hand, #0 end-stage renal disease was an important risk factor in patients with COVID-19. Patients with end-stage renal disease (ESRD) have weaker immune systems and frequent hospital visits, which may explain why they are more susceptible to COVID-19 infection than the general population^20^. Clinical studies have found that the mortality rate of COVID-19 patients with ESRD was 38%^20^. Even after adjusting for factors such as complications, ESRD was still significantly associated with the high mortality rate of COVID-19^20^. On the other hand, #4 acute kidney injury was a common complication of COVID-19 and an independent risk factor for death in patients with COVID-19^21^. Severe COVID-19 disease is often complicated by acute kidney injury, which may transition to CKD^15^.

Both #1 cardiovascular disease and #3 diabetes mellitus had an important place in the course of COVID-19. Cardiovascular disease (CVD) was a common complication of COVID-19, and individuals with cardiovascular disease were also more likely to be infected with COVID-19^22^. The most common cardiovascular complication in COVID-19 patients was myocardial injury (21.2%), followed by arrhythmias (15.3%) and heart failure (14.4%)^23^. However, myocardial injury and heart failure were most common in patients who died^23^. There was a bidirectional interaction between diabetes mellitus (DM) and COVID-19: DM increased the rate of severe disease and mortality in COVID-19, while COVID-19 promoted the progression of DM^24^. Moreover, DM in patients with COVID-19 infection was associated with a 0.549-fold increase in mortality^25^. Although CVD and DM were identified as high-risk factors for COVID-19 earlier than CKD, CKD had a more significant impact on mortality^14,26^.

As we all know #2 kidney transplantation is an effective treatment for CKD, and the COVID-19 pandemic has greatly impacted the field of kidney transplantation. Transplant policies, donor chains, and timely and safe transplants were negatively impacted during the outbreak^27^. Due to immunosuppressive status and comorbidities, kidney transplant patients with COVID-19 have a higher rate of acute kidney injury and death than the next person^13^. A meta-analysis of 5559 COVID-19-positive kidney transplant recipients revealed a 23% mortality rate and a 50% incidence of acute kidney injury^28^. Before the COVID-19 pandemic, #6 convalescent plasma had been used in the treatment of other viral infections with positive results^29^. For kidney disease patients who are unable to utilize antiviral medications due to contraindications or safety concerns, convalescent plasma is an alternate treatment^30^. Unfortunately, multiple studies have found that convalescent plasma had no impact on the incidence of mortality or composite poor outcomes in COVID-19 patients^31–33^.

#5 Meta-analysis is a commonly used and effective statistical method. Of the 622 publications included in this study, there were 50 meta-analyses. These 50 publications not only provide high-quality clinical evidence but also study and solve many clinical problems related to COVID-19 and CKD.

Based on the above hot spots, we speculated that the research frontier in this field may focus on solving clinical problems. COVID-19 patients with CKD not only have a worse prognosis but are also more likely to develop acute respiratory distress syndrome or pneumonia^34^. Although a lot of research has been conducted to find a solution for COVID-19, satisfactory results have not been achieved^30^. Therefore, how to help CKD patients, especially ESRD patients, prevent COVID-19 is very important for both doctors and patients. CKD patients should take all necessary measures to avoid SARS-CoV-2 infection. Medical institutions and scientific research institutions should also accelerate research in this field, such as reducing the risk of infection in patients undergoing dialysis and kidney transplantation, and developing a COVID-19 vaccine suitable for ESRD patients.

### Limitations

This study had some limitations. Firstly, owing to CiteSpace’s format constraints, we only counted publications in the WoSCC database, which may have disregarded papers only in other databases such as PubMed, Embase, and Cochrane Library. However, the professional authority of the WoSCC database and the widespread cross-replication of records in other databases mean that this study can still be used to show the general state of affairs and general trends in this field. Secondly, only English publications were included in the study, which might have influenced the results due to source bias. As researchers globally are currently still accustomed to publishing important findings in English-language journals, the inclusion of English publications alone would not have a significant impact on the research results.

## Conclusion

Although the world is gradually recovering from the disasters caused by COVID-19, COVID-19 still affects our lives. Currently, people are conducting more extensive and in-depth research on COVID-19. The USA, China, and some European countries, as well as their research institutes, have demonstrated robust research capacity and research leadership. However, African countries are clearly lagging behind in research progress in this area, and they need more cooperation and assistance.

ESRD, CVD, DM, acute kidney injury, kidney transplantation and convalescent plasma are hot topics and research frontiers in the field and represent, to some extent, future trends. We see clinical practice as a future focus for the field and have raised a number of issues that need to be addressed in the discussion section.

## Ethical approval

This article does not deal with original human or animal data. Ethical approval is not applicable.

## Consent

This article does not involve research on patients or volunteers. Consent is not applicable.

## Source of funding

none.

## Author contribution

J.W. and C.Y. designed the study and wrote the manuscript. Z.W., Q.E., and H.Y. collected the data. Z.Y. and G.Y. re-examined the data. J.W. and C.T. analyzed the data. L.K. reviewed and revised the manuscript. All authors contributed to the article and approved the submitted version.

## Conflicts of interest disclosure

None.

## Research registration unique identifying number (UIN)

1. Name of the registry: Not Applicable.
2. Unique Identifying number or registration ID: Not Applicable.
3. Hyperlink to your specific registration (must be publicly accessible and will be checked): Not Applicable.

## Guarantor

Keda Lu.

## Data availability statement

The data used in this study are available on reasonable request from the first and corresponding authors.

## Provenance and peer review

None.
